# Peers plus mobile app for treatment in HIV (PATH): protocol for a randomized controlled trial to test a community-based integrated peer support and mHealth intervention to improve viral suppression among Hispanic and Black people living with HIV

**DOI:** 10.1186/s13063-024-08042-8

**Published:** 2024-03-22

**Authors:** Eileen V. Pitpitan, Keith J. Horvath, Jeannette Aldous, Jamila K. Stockman, Thomas L. Patterson, Megan Liang, Constantino Barrozo, Veronica Moore, Katherine Penninga, Laramie R. Smith

**Affiliations:** 1 School of Social Work, San Diego State University, San Diego, USA; 2 Division of Infectious Diseases and Global Public Health, University of California San Diego, La Jolla, USA; 3 Department of Psychology, San Diego State University, San Diego, USA; 4 San Ysidro Health, San Diego, USA; 5 Department of Psychiatry, University of California San Diego, La Jolla, USA; 6 San Diego State University Research Foundation, San Diego, USA

**Keywords:** HIV care, ART, Viral suppression, Intervention, Peer navigation, mHealth, Implementation, Randomized controlled trial, Hispanic, Black

## Abstract

**Background:**

Significant disparities continue to exist in the HIV care continuum, whereby Hispanic and Black people living with HIV (PLWH) are less likely to achieve viral suppression compared to their White counterparts. Studies have shown that intervention approaches that involve peer navigation may play an important role in supporting patients to stay engaged in HIV care. However, implementation may be challenging in real-world settings where there are limited resources to support peer navigators. Combining a peer navigation approach with scalable mobile health (mHealth) technology may improve impact and implementation outcomes.

**Methods:**

We combined a peer navigation intervention with a mHealth application and are conducting a randomized controlled trial (RCT) to test the efficacy of this integrated “Peers plus mobile App for Treatment in HIV” (PATH) intervention to improve HIV care engagement, and ultimately sustained viral suppression, among Hispanic and Black PLWH. We will enroll up to 375 PLWH into a two-arm prospective RCT, conducting follow-up assessments every 3 months up to 12 months post-baseline. Participants randomized to the control arm will continue to receive usual care Ryan White Program case management services. Individuals randomized to receive the PATH intervention will receive usual care plus access to two main intervention components: (1) a peer navigation program and (2) a mHealth web application. The primary outcome is sustained HIV viral suppression (undetectable viral load observed at 6- and 12-month follow-up). Secondary outcomes are retention in HIV care, gaps in HIV medical visits, and self-reported ART adherence. Recruitment for the RCT began in November 2021 and will continue until June 2024. Follow-up assessments and medical chart abstractions will be conducted to collect measurements of outcome variables.

**Discussion:**

The efficacy trial of PATH will help to fill gaps in our scientific understanding of how a combined peer navigation and mHealth approach may produce effects on HIV care outcomes while addressing potential implementation challenges of peer navigation in Ryan White-funded clinics.

**Trial registration:**

The PATH trial is registered at the United States National Institutes of Health National Library of Medicine (ClinicalTrials.gov) under ID # NCT05427318. Registered on 22 June 2022.

## Administrative information

**Table:** 

Title {1}	Peers plus mobile app for treatment in HIV (PATH): Protocol for a randomized controlled trial to test a community-based integrated peer support and mHealth intervention to improve viral suppression among Hispanic and Black people living with HIV
Trial registration {2a and 2b}	The PATH trial is registered at the United States National Institutes of Health National Library of Medicine (ClinicalTrials.gov) under ID # NCT05427318
Protocol version {3}	Protocol Version 1.0
Funding {4}	Funding for this trial was provided by a grant funded from both the United States National Institute of Drug Abuse and National Institute of Nursing Research (R01DA053167; PI: Pitpitan). Preparation of this manuscript was supported by the San Diego Center for AIDS Research, funded by the United States National Institutes of Health (P30AI036214)
Author details {5a}	Eileen V. Pitpitan^1, 2^ Keith J. Horvath^3^ Jeannette Aldous^4^ Jamila K. Stockman^2^ Thomas L. Patterson^5^ Megan Liang^6^ Constantino Barrozo^4^ Veronica Moore^4^ Katherine Penninga^4^ Laramie R. Smith^2^ ^1^School of Social Work, San Diego State University, San Diego, US ^2^Division of Infectious Diseases and Global Public Health, University of California San Diego, La Jolla, US ^3^Department of Psychology, San Diego State University, San Diego, US ^4^San Ysidro Health, San Diego, US ^5^Department of Psychiatry, University of California San Diego, La Jolla, US ^6^San Diego State University Research Foundation
Name and contact information for the trial sponsor {5b}	National Institute on Drug Abuse C/O NIH Mail Center 3WFN MSC 6024 16,071 Industrial Dr—Dock 11 Gaithersburg, MD 20892* *(Use 20,892 for U.S. Postal Service, 20,877 for UPS and FedEx)
Role of sponsor {5c}	The sponsor provided funding for this trial, and is not responsible for the study design, collection, management, analysis, data interpretation. The sponsor did not contribute to the decision to submit this manuscript for publication

## Introduction

### Background and rationale {6a}

Significant disparities continue to exist in the HIV care continuum, whereby Hispanic and Black women and men living with HIV are less likely to achieve viral suppression compared to White women and men living with HIV [1–7]. In 2016, among people living with HIV (PLWH) aged 13–29, an estimated 42% of Blacks/African Americans and 36% of Hispanics in the US were not virally suppressed [8]. Disparities are exacerbated by co-occurring syndemic conditions, including substance use and mental health conditions [9–11]. As elsewhere, substance use is a robust correlate of HIV infection in San Diego [12, 13], especially near the Mexico border where methamphetamine and opioids are trafficked and readily accessible [14]. Therefore, interventions to promote viral suppression among PLWH must be developed and tested in the most vulnerable, lower-resourced communities, including communities affected by substance use.

Community health workers, and particularly peer navigators (also referred to as peers, “promotoras/es,” or “compañeros/as de apoyo”), have been shown to play meaningful roles in supporting patients living with HIV to stay engaged in care in developing countries [15, 16]. The evidence base is smaller in the US. A 2016 systematic review of interventions involving PLWH serving as peers to improve HIV care continuum outcomes identified nine published studies [17]. Only a minority (44%) were in the US with the rest (56%) in sub-Saharan Africa. While six of the nine used a randomized controlled trial (RCT) design, only one study found a positive effect on viral suppression. Further, only two of the four interventions in the US were tested with a sample who were predominantly racial/ethnic minority, but neither specified addressing barriers to HIV care unique to Hispanic and Black PLWH (i.e., stigma, medical mistrust). The findings from this review suggest that more studies applying a rigorous design are needed to evaluate the impact of peer navigation on HIV care continuum outcomes in the US, particularly to address barriers unique to racial/ethnic minorities living with HIV [18–20].

While peer navigation may promote positive health outcomes among PLWH, implementation and scale-up of this approach may encounter difficulties in real-world clinical and community settings where there are limited resources to support peer navigation programs [21, 22]. In contrast, mobile health (mHealth) is a scalable approach that can be more feasibly implemented since it requires fewer staff resources [23, 24]. To further illustrate, a clinic may require a large team of peer navigators to adequately support an entire caseload of patients with HIV. However, when combined with mHealth, the impact that peer navigators can have on supporting people with HIV may be strengthened. Thus, combining peer navigation and mHealth intervention approaches may be one strategy to simultaneously reduce the need for larger teams of peer navigators, while also strengthening the impact of peer navigation on HIV care continuum outcomes among Hispanic and Black PLWH. mHealth and other technologies offer platforms for intervention delivery through various devices, such as laptops, tablets, smartphones, and channels, such as online, mobile apps, SMS, and social networking platforms, to overcome the limitations and costs of in-person interventions [23, 24]. In a systematic review of mHealth interventions designed for racial/ethnic minority groups, those that focused on HIV were aimed at HIV primary prevention, with only two focused on HIV care self-management, with text message reminders to support antiretroviral therapy (ART) adherence [25]. Also, a review of mHealth interventions to support self-management in HIV found that of the 28 interventions identified, the majority (57%) used text messaging, and only 5 (18%) used mHealth applications (apps) [26]. Of the five mobile app interventions, none appeared to be tailored to address challenges, such as stigma and substance use [26], experienced by Hispanic and Black PLWH. Altogether, the evidence suggests that there is a critical absence of rigorous, randomized controlled trial (RCT) studies in the US to evaluate the efficacy of peer navigation and mHealth intervention approaches to improve viral suppression and retention in HIV care among Hispanic and Black PLWH.

Combining both peer navigation and mHealth technology into a unified, scalable intervention could both strengthen the impact of peer navigation on HIV care outcomes among PLWH and simultaneously reduce the amount of resources needed to support peer navigation in clinical settings, effectively striking the needed balance between clinical effect sizes and implementation costs [27]. In this trial, we propose the Peers plus mobile App for Treatment in HIV (PATH) intervention that may serve as an integrated solution that builds on the strengths of peer navigation to improve HIV care engagement and ART adherence, while also addressing potential implementation challenges of peer navigation.

## Objectives {7}

The primary goal of this RCT is to evaluate the efficacy of the PATH intervention compared to usual care in a community-based HIV care setting on our pre-defined primary and secondary outcomes. Specific aims are threefold: (1) examine efficacy of PATH on primary (viral suppression at 6 and 12 months) and secondary outcomes (retention in HIV care; gaps in HIV medical visits; self-reported ART adherence); (2) examine the theory-informed mediators (e.g., increased self-efficacy to engage in HIV care, and reduced medical mistrust and HIV stigma), through which PATH may have the greatest impact on outcomes among Hispanic and Black PLWH; and (3) among those using substances, explore whether PATH significantly affects substance-related outcomes, including frequency of substance use and engagement in substance use treatment, when compared to usual care (UC). A sub-aim of the trial is to explore subgroup differences in efficacy based on factors such as race/ethnicity and substance use.

## Trial design {8}

We are implementing a two-arm parallel group randomized controlled superiority trial design, whereby enrolled participants complete a baseline assessment, and then are randomized on a 1:1 ratio into one of two arms. In the control arm, participants receive UC Ryan White coordinated case management services (i.e., standard intake and as needed, referrals medical, dental and mental health services, case management, and alcohol/substance use treatment]). In the PATH intervention arm, participants receive UC plus two main intervention components: (1) support from a peer navigation program and (2) access to the PATH mHealth web application. Post-baseline/randomization, participants are asked to complete follow-up assessments at 3, 6, 9, and 12 months.

## Methods: participants, interventions, and outcomes

### Study setting {9}

The RCT is being implemented in a community-based federally qualified health center (FQHC) located in the southern part of San Diego County, CA, along the US-Mexico border. This FQHC offers HIV primary and sub-specialty medical services and coordinated case management services at multiple locations to a majority racial minority population, which will help achieve the study goal of reaching Hispanic and Black PLWH.

### Eligibility criteria {10}

Individuals are eligible to participate in PATH if they meet the following criteria: (a) are at least 18 years old; (b) can read and speak English or Spanish; (c) are living with HIV and at least 3 months have passed since HIV diagnosis; (d) currently prescribed antiretroviral therapy (ART) medication; (e) have regular access to an Internet browser on a computer, tablet, or smartphone; (f) do not plan to move out of the San Diego area in the next 12 months; (g) not currently enrolled in any other program, intervention, or research study designed to improve HIV adherence or engagement in HIV care; and (h) not currently a member of one of the community advisory boards providing advisement on the study. In addition to criteria *a* through *g*, eligible participants must meet one or more of the following criteria: (1) one or more detectable VL test result (> 200 copies m/L) in the past 12 months while on ART for at least 3 months; (2) missed 1 or more scheduled HIV care appointments in the last 12 months; (3) last HIV care visit with an HIV care provider was more than 6 months ago; (4) report anything less than *excellent* adherence on one of the Wilson-3 ART adherence scale [28] items; and/or (5) report non-prescription stimulant or opioid use in the past 6 months. The first three indicators are verified using medical chart information, and the last two are assessed via self-report on the screening questionnaire. Finally, since the intervention was designed in a manner whereby the PATH peer navigators are meant to provide support as part of the HIV care team at the community-based FQHC, and as a study that is interested in exploring implementation outcomes of the PATH intervention, eligible participants are required to be current HIV medical care patients of the community-based FQHC.

### Who will take informed consent? {26a}

Written informed consent is gathered from participants. During the enrollment visit, trained research staff explain the study procedures, risks, and strategies to minimize risk during the informed consent process.

### Additional consent provisions for collection and use of participant data and biological specimens {26b}

In addition to providing informed consent, enrolled participants are required to provide permission for the researchers to use their personal health information for the study. Specifically, participants are asked to authorize the release of their HIV/AIDS treatment information (i.e., lab reports of HIV viral load, HIV care medical visit dates) from the community-based FQHC for the research.

### Interventions

#### Explanation for the choice of comparators {6b}

The comparator in this trial is the community-based FQHC’s existing Ryan White medical and coordinated case management services as usual. Their UC includes outpatient/ambulatory medical care, AIDS Drug Assistance Program (e.g., through Medicare, Medi-Cal), oral healthcare, early intervention services, mental health services, health education, medical nutrition therapy, medical case management, substance use disorder outpatient services, and support services (e.g., emergency financial assistance, food pantry, housing services, medical transportation, and legal services).

#### Intervention description {11a}

The PATH intervention integrates two theoretically grounded interventions developed by the team of investigators—a peer navigation intervention and an mHealth tool—with the goal of amplifying the impact of peer navigation on viral suppression among Hispanic and Black PLWH.

The peer navigation component is informed by our prior research developing “Conexiones Saludables” (“Healthy Connections” in Spanish; “Conexiones” for short), which is a theory-based intervention including modularized training for peer navigators to build core competencies in supporting marginalized PLWH [29, 30]. Peer navigators undergo 3 weeks of daily modularized training (15 modules total). Conexiones also included peer-delivered “peer empowerment sessions” designed to promote information, motivation, and behavioral skills (self-efficacy) in HIV care engagement and ART adherence, especially in the context of co-occurring conditions like substance use and mental health conditions. Sessions are conducted on a one-on-one basis between the participant and their assigned peer navigator and are delivered monthly over approximately 6 months using motivational interviewing techniques to facilitate intrinsic motivation for behavior change [31]. The design of Conexiones was based on the Situated Information, Motivation, and Behavioral skills (IMB) Model [32], which outlines how behavioral skills to engage in HIV care and adhere to ART should be situated to one’s specific socio-structural context, such as substance use or cultural norms in order to enact behavior change (e.g., ART adherence) [32, 33]. For example, providing information about ART adherence strategies aligned with drug use patterns, understanding sociostructural factors, such as intersectional stigma and discrimination, affecting motivation to engage in healthcare and treatment, and developing behavioral skills, such as self-efficacy, to maintain ART adherence even in a context of substance use stigma in the healthcare setting. In addition to the Situated-IMB Model, Conexiones was also founded in the Theory of Triadic Influence [34], a multilevel Social Cognitive theory [35] that hypothesizes three “streams of influence” (individual, social, and structural) which may act simultaneously to affect self-efficacy to engage in healthy behavior. Operationalized to the health behavior context of Hispanic and Black PLWH, the streams of influence most relevant to PATH include individual-level barriers (being a racial/ethnic minority, substance use) and socio-structural level barriers, such as or substance use stigma or medical mistrust, that decrease both Hispanic and Black people’s engagement in health and HIV care [18–20, 36–39]. Conexiones pilot data demonstrated improvement in HIV care continuum outcomes among marginalized Hispanic PLWH, such as people who use and/or inject drugs, in the US-Mexico border region [29, 30, 40].

The mHealth component of PATH is informed by “LinkPositively,” a mobile-optimized web application (or “webapp”) aimed at improving engagement in the HIV care continuum [41]. The LinkPositively intervention includes interactive features that were based on the IMB model [32, 42]. For example, the webapp incorporated information relevant to HIV self-management (educational and self-care tips feature), motivational enhancements (social support via a feed that participants can post to and reply to comments from other participants and gamification features), and behavioral skills (ART self-monitoring feature) to improve ART adherence. Based on focus group and community feedback, adaptations were made to the original Conexiones and LinkPositively interventions to make them more culturally tailored for both Hispanic and Black PLWH (e.g., creating English and Spanish language versions, using images that both Hispanic and Black participants may identify with).

**Fig. 1:**
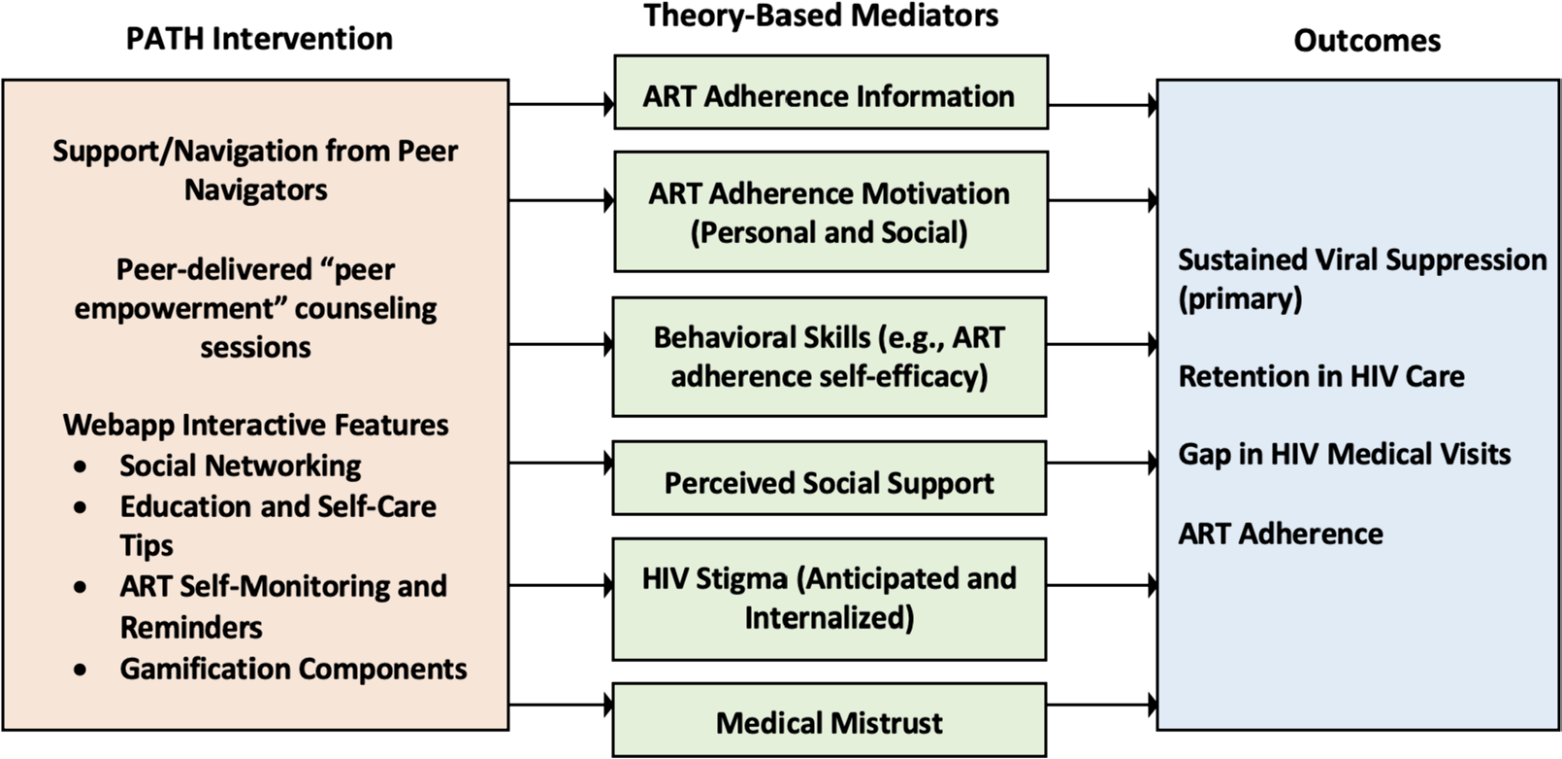
Summary of PATH intervention features and the theory-informed mechanisms of action that are hypothesized to produce effects on the primary and secondary outcomes

Applying the theoretical foundations that informed the PATH peer navigation and mHealth components, Fig. 1 summarizes how PATH is hypothesized to target multiple psychological and social mechanisms of action, which should ultimately lead to a potent impact on HIV care outcomes among Hispanic and Black PLWH. Overall, participants assigned to the PATH intervention arm are assigned to receive support from one peer navigator and have access to the PATH webapp. Peer navigators meet with their clients at least monthly to deliver peer empowerment sessions. Empowerment sessions occur during the first 6 months, after which participants continue to have access to their peer and the PATH webapp for 12 months total, for example by messaging the peer through the PATH webapp. As much as possible, the study aims for peers to reflect the patient population by recruiting peers who speak English and Spanish, and who are Hispanic or Black. The PATH webapp is available in both languages. Translations of the webapp content were first translated then back-translated and reviewed for cultural and linguistic accuracy.

#### Criteria for discontinuing or modifying allocated interventions {11b}

The outcome analysis will follow the intent-to-treat approach. Under no circumstances is it possible for the allocated intervention (i.e., usual care or PATH intervention) to be modified once the participant has been enrolled in the trial. However, participants may be unenrolled from the study at any point due to voluntary withdrawal for any reason.

#### Strategies to improve adherence to interventions {11c}

For participants randomized to the PATH intervention arm, a number of strategies are used to promote adherence to the intervention. These include intensive one-on-one training on how to use the PATH webapp and its features, including re-training upon request. Upon randomization to the intervention arm, participants are also asked to sign a client confidentiality agreement which outlines how PATH intervention participants can expect to receive professional, timely, respectful, and trustworthy peer support. Finally, whenever possible, all clients receive a “warm handoff” to immediately meet their assigned peer navigator over Zoom or in person following randomization into the PATH arm.

#### Relevant concomitant care permitted or prohibited during the trial {11d}

One of the study eligibility criteria is that participants must not currently be enrolled in any other program, intervention, or research study designed to improve HIV adherence or engagement in HIV care (regardless of intervention approach). While this is assessed during screening procedures, if a participant were to receive HIV care and/or ART adherence support from another program or research study during their participation in the trial, this information is recorded during follow-up assessments but does not require disenrollment from the trial.

#### Provisions for post-trial care {30}

Participants will not be provided any additional care outside of UC from the community-based FQHC.

### Outcomes {12}

The primary outcome in this trial is sustained viral suppression, defined as an HIV viral load at 6 and 12 months post-enrollment of less than 200 copies/mL. Secondary outcomes include the following variables: *Retention in HIV care* (at 6- and 12-month follow-up) is assessed through chart review using the HRSA definition of having greater than or equal to 2 HIV medical visits at least 90 days apart within the 12-month follow-up. *Gap in HIV medical visits* is measured through chart review at 6 and 12 months using the HRSA definition (no HIV medical visits in the last 6 months) [43]. *ART adherence* is measured using the self-report Wilson-3 scale [28] at 3-, 6-, 9-, and 12-month follow-up. The secondary outcomes were chosen to fill gaps in outcome measurements in the HIV peer navigation intervention literature [17] and to have a broader representation of the stages of engagement in the HIV care continuum [4].

### Participant timeline {13}

**Fig. 2:**
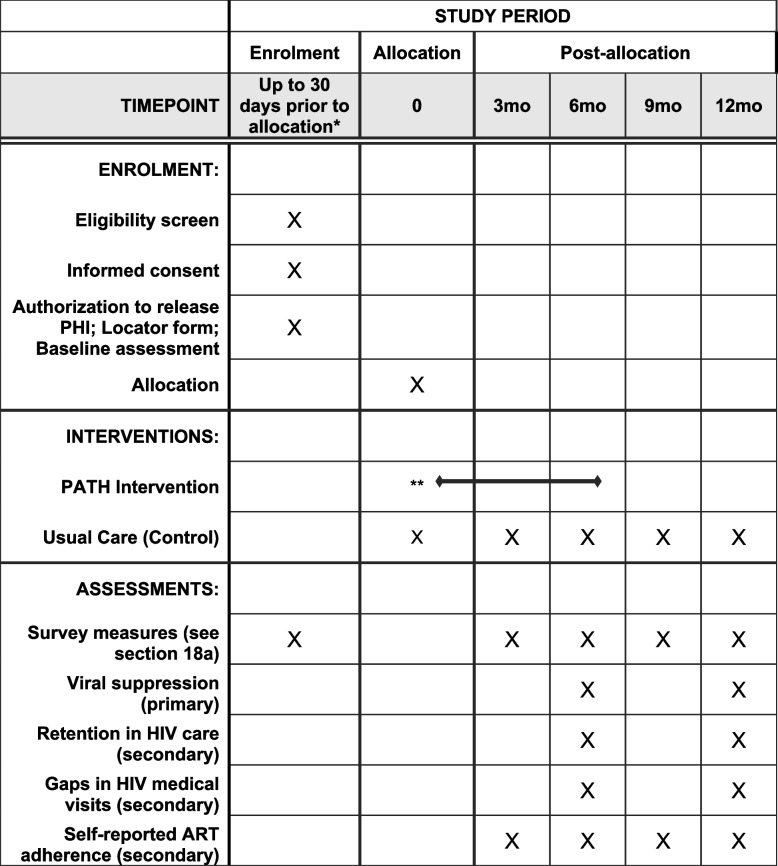
Schematic of participant timeline in the PATH RCT. *Participants are allowed to finish enrollment activities up to 30 days after they initially screen for eligibility, after which point they are required to re-screen. **Participants who are randomly allocated to the PATH intervention arm immediately complete intervention onboarding activities, which include assignment to their PATH peer navigator, creation of their account for the PATH web application, and in-depth tutorial on how to log into the PATH app and its features

Interested participants meet with study staff (in person or over the phone) to complete a screening questionnaire. Once eligibility criteria are verified by study staff, the participant is asked to complete enrolment procedures within 30 days of completing screening. If 30 days have passed since the screening, the participants must be re-screened again to verify that they continue to be eligible for the study. Study enrolment procedures consist of the informed consent process, authorization to release personal health information for the research, completing a “locator” form to share details for future contact with the participants, and completing the baseline assessment through self-administration via an online survey tool (Qualtrics, Provo, UT). Only after these procedures have been completed, including the baseline assessment, are participants officially enrolled in the study and randomly assigned to either the control or the PATH intervention arm. Assignment to study arm is concealed to both study staff and participants until this final stage. A schematic of the participant timeline in the RCT is shown in Fig. 2.

### Sample size {14}

**Fig. 3:**
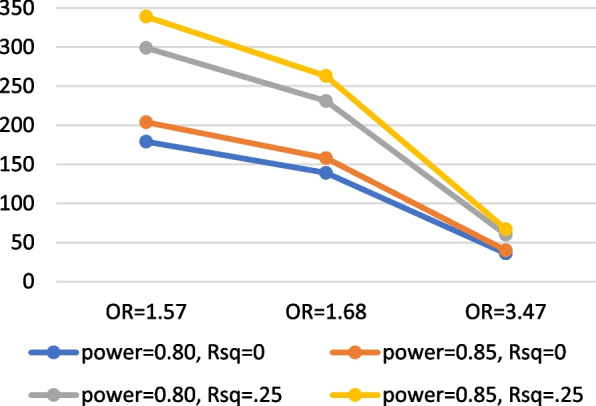
Power and sample size estimates based on small, medium, and large intervention effect sizes and covariates

It was estimated that a sample size of up to 375 total participants would be required to achieve study objectives. This estimate was informed by a sample size calculation. Figure 3 displays power and sample size estimates based on intervention effect sizes and covariates. Meta-analytic data from Finitsis et al. [44] of mHealth text messaging interventions to promote ART adherence among PLWH has shown that such interventions can improve VL suppression (*k* = 3; OR = 1.57, 95% CI = 1.11, 2.20). This OR of 1.56 is a small effect size, and Chen et al. [45] established conventions of small, medium, and large ORs (similar to Cohen’s magnitudes of effect size, *d*). Based on the Finitsis meta-analysis (OR = 1.57), and accounting for other possible effect sizes of small (OR = 1.68) or medium (OR = 3.47) magnitude (based on Chen et al.) [45], the required sample sizes to achieve adequate power of at least 0.85 ranges from *n* = 179 to 204; with covariates in the model (accounting for 25% of the variance in the outcome, then a sample size ranging from *n* = 263 to 339 would be required). To provide further support for this power analysis, we conducted another analysis in G*Power using an effect size estimate from a recent peer navigation intervention (that does not include a mHealth component, “LINK LA” [46]) for HIV-positive men and transgender women released from jail. In this study, the investigators tested the efficacy of their peer navigation intervention compared to usual care (case management) and found a 22% difference-in-difference between the intervention and control arm on viral suppression over a 12-month follow-up. Using this effect size information, and assuming 0.80 power, alpha = 0.05, and 1:1 allocation ratio between intervention and control arms, the required sample size is 97 per group, or a total *n* of 194. Altogether, our calculations to estimate the desired sample size with adequate power and, based on up to 20% anticipated attrition, a sample size between 225 and 375 would provide us with adequate power to achieve our aims. Thus, our goal is to randomize up to 375 participants for an analytic sample size of ~ 300 (150 per arm).

### Recruitment {15}

Participants are recruited internally within the community-based FQHC. The clinic data team regularly creates, updates, and shares a list of potentially eligible patients based on their recent HIV care history with the research team. Clinic staff also help to recruit from their patient population by reviewing clinic schedules weekly to identify participants with a pending appointment who might qualify based on the eligibility criteria for on-site recruitment. Other clinic staff, such as case managers and general medical staff, are informed of study details and eligibility to provide preliminary study information and refer potential participants. Research staff also work with other teams within the HIV Department to promote the study. The clinic’s community advisory board also serves as a resource for informing potential participants that the study is occurring, and study participants can also refer friends. When a patient is identified to be potentially eligible for the study (e.g., through clinic staff referral, or by reviewing clinic data), study staff attempt to recruit the individual, usually by phone. Study staff introduce the study and provide the next steps for interested patients. Those not interested are given an opt-out option to limit further contact from the team.

## Assignment of interventions: allocation

### Sequence generation {16a}

Enrolled participants are randomized on a 1:1 basis into the PATH intervention or usual care. Randomization is determined using an online generated randomization schedule determined prior to the study launch (a priori). Randomization is not stratified by substance use or race, although recruitment efforts are made to help ensure that at least a third of the sample are people with active substance use (defined as using non-prescription stimulants or opioids in the past 6 months) and that the sample is representative of the HIV patient population at the community-based FQHC (i.e., at least 60% Hispanic).

### Concealment mechanism {16b}

Once a participant completes enrolment procedures, a research staff member (peer navigators are not research staff) completes an online enrollment verification and randomization form (via Qualtrics). The form was pre-programmed by the PI and Study Coordinator in such a way that the study arm is revealed to the staff member once the participant ID is assigned and entered. Participant ID number determines study arm assignment (as defined by the online generated randomization schedule). This method ensures that both the study arm and sequence of assignments are concealed until conditions are assigned.

### Implementation {16c}

The allocation sequence was determined randomly using an online generated randomization scheduling tool, and study staff are trained to enroll participants in their preferred language (English or Spanish).

## Assignment of interventions: blinding

### Who will be blinded {17a}

Enrolled participants are not blind to their study assignment (they know if they are assigned a peer navigator and have access to the PATH web app or not), and neither are peer navigators nor HIV care providers. Staff who complete chart abstraction for outcome assessment and data analysts are blinded to study arm assignment.

### Procedure for unblinding if needed {17b}

The design is an open label with only outcome assessors and data analysts being blinded so unblinding will not occur.

## Data collection and management

### Plans for assessment and collection of outcomes {18a}

To measure the primary outcome, sustained viral suppression, viral load data will be collected through medical chart review (within a 30-day window of when the 6- and 12-month follow-ups are due). Participants who do not have a viral load in the chart within the 30-day (pre/post) window are invited to go to any Quest lab location for a blood draw to collect viral load information for study purposes. For participants randomized into the PATH intervention arm, peer navigators offer reminders of when labs are due as part of usual HIV care. For participants randomized into the control arm, if a study-related lab is overdue, then they are notified by research staff at the community-based FQHC and encouraged to complete a blood draw at a Quest lab. Secondary outcomes, retention in HIV care and gaps in HIV medical visits, will be assessed through medical chart review. Self-reported ART adherence will be measured using the Wilson-3 scale [28], a continuous measure of ART adherence (0–100; 100 = perfect adherence).

Other trial data measured at baseline and follow-up include sociodemographic information (e.g., age, gender, race, sexual orientation, years living with HIV), as well as validated measures on ART adherence-related information, motivation, and behavioral skills [42, 47], eHealth literacy [48], technology use [49], rapport with an assigned peer navigator, HIV-related stigma [50], social support [51], medical mistrust [52], depressive symptoms [53], PTSD [54], experiences with violence [55, 56], food security [57], housing instability [58], substance use [59], substance use-related stigma [60], and substance use treatment history.

### Plans to promote participant retention and complete follow-up {18b}

A study coordinator who is based on-site at the community-based FQHC is responsible for overseeing study retention/follow-up survey collection. Strategies to promote study retention include the use of a comprehensive “locator form” completed during the enrolment process, whereby each participant is asked about various and preferred methods (e.g., phone, email, text) they may be contacted to allow for staff to send follow-up survey reminders. Staff are trained to update the locator form during contact with the participant at each follow-up assessment. The study coordinator attempts to contact participants up to 2 weeks before the next follow-up survey is due and continues to attempt contact through various methods until 45 days after the follow-up is due (after which point the visit is considered missing). In addition, the study coordinator visits clinic locations on a regular basis to provide on-site support to participants who may prefer to complete their survey on-site at the community-based FQHC (accessing the survey on Qualtrics through an office computer or tablet).

### Data management {19}

All data for this trial are collected via an online survey platform, Qualtrics (Provo, UT; First released in 2005; version used was current at the time of study activities). Baseline and follow-up surveys are self-administered by participants, although study staff are available at all times to answer any questions that might arise during survey completion. Case report forms, including medical chart abstraction data, are entered by trained study staff. Qualtrics complies with applicable data privacy laws in its role as a data controller of its own data and as a data processor of customer data. All data are backed up by Qualtrics using two methods: automatic propagation across servers (immediate upon collection) and daily complete off-site encrypted backups. In addition, the study’s project manager conducts monthly back-ups of all data. Data managers are responsible for examining frequencies for categorical variables (e.g., education) and measures of central tendency for continuous variables (e.g., age). Range checks were defined, a priori, for every variable, and out-of-range values are automatically flagged and reported in reports compiled semi-annually. This process assists with the detection of missing data prior to outcome analysis.

### Confidentiality {27}

This trial takes precautions to protect participants’ confidential information (for example, HIV status and drug use). Participants are only identified in the study by a participant ID number. All data collected from recruited and enrolled participants, including electronic data, are identified only by the participant’s study ID and are protected against access by anyone except authorized staff connected to the study. Any hard copy data are stored in locked cabinets in secure offices, while electronic data are password-protected. HIV status or illicit drug use information does not appear along with any personal identifying information such as the locator form. The locator form containing participants’ contact information is completed online over Qualtrics. The computer file that contains the key to participants’ code numbers (name-to-ID relational file) is encrypted, and the computers on which the file resides are locked in the study coordinator’s office. Only authorized PATH trial staff are able to remove encryption from the computer file. Other security mechanisms include security workshops and written security policies and procedures. For example, all study staff are required to receive certification in “Information, Privacy, and Security” training, offered via the Collaborative Institutional Training Initiative (CITI) program. Protections against breaches of confidentiality are also implemented on the PATH webapp. Specifically, user profiles are monitored by study staff and protected from personal identifying information, to maintain the participant’s confidentiality, and all other features of the webapp are monitored in the same way. Finally, participant responses on surveys remain confidential and are not shared with their medical care providers or staff at the community-based FQHC. Once all data have been collected for the trial, the datasets will de-identified prior to analysis.

### Plans for collection, laboratory evaluation, and storage of biological specimens for genetic or molecular analysis in this trial/future use {33}

Biological specimens for HIV viral load are not being directly collected by the researchers of this trial. Instead, HIV viral load data will be abstracted from medical record (chart) information already being collected as part of UC at the community-based FQHC, or through abstraction of Quest lab results (as described in Item 18). Relatedly, the trial is not conducting any laboratory evaluation or storage of any other biological specimens.

## Statistical methods

### Statistical methods for primary and secondary outcomes {20a}

Following CONSORT guidelines, potential imbalance across the two arms on potential confounders will be examined. If preliminary analyses detect non-trivial imbalance that cannot be satisfactorily addressed via covariate adjustment, we will substitute causal inference methods using propensity score weighting or marginal structural models [61–65] to obtain the effects of the intervention.

The primary analytic approach will utilize generalized mixed-effects models and an intent-to-treat (ITT) approach. This addresses issues with attrition, missing data, and other unintended violations of design, thereby allowing us to maximize statistical power by not excluding subjects with some missing data. Mixed-effects models incorporate random intercepts and slopes for each participant based on the participant’s multiple measurements over time [65]. Intra-class correlations among participants nested within specific peer navigators will be examined to diagnose whether significant variation in outcomes is explained by the shared peer navigator, if so, models will account for clustering. Attrition analyses will compare participants who complete all measurements to those who do not based on baseline characteristics. If there is a baseline imbalance between participants retained in the study versus not across certain characteristics, then these will be accounted for in all analyses. In addition, maximum likelihood will be used to address incomplete data because this makes the relatively mild assumption that missing data are conditionally missing-at-random [66]. Outcome analysis will apply a priori planned comparisons. It is hypothesized that compared to control, participants in the PATH intervention will exhibit the following: greater viral suppression at 6- and 12-month follow-up (HYP 1); greater retention in care (HYP 2); fewer gaps in HIV care (HYP 3); greater ART adherence (HYP 4). HYP 1–3 involve binary outcomes (Y/N on sustained viral suppression, retained in care, had gaps in care), while HYP 4 is a continuous outcome (proportion). These hypotheses will be tested using regression analysis specifying the appropriate outcome distribution (e.g., binomial) and function (e.g., logit), with *α* = .05; any subsequent post-hoc comparisons will be adjusted via simulation-based stepdown methods [67] to maintain a nominal type 1 error rate of .05. For the primary outcome sustained viral suppression, we will assume non-suppression at that time point (6 or 12 months) for any missing viral load chart data. A sensitivity analysis will be conducted utilizing only available viral load chart data to compare with the results assuming missing viral load equals non-suppression.

### Interim analyses {21b}

Interim analyses are completed to monitor sample characteristics across screening data (e.g., the proportion of participants who meet one or more indicators for falling out of HIV care, as described in the section summarizing eligibility criteria), and to conduct a range of checks on the data, and detect for potential invariance across the two study arms. The study investigators have access to these interim results. No stopping guidelines have been formally outlined in the protocol, but the trial does apply a detailed plan to monitor and address potential and serious adverse events, which include timely communication with the study IRB and study sponsor (NIH), who may decide to terminate the trial if needed.

### Methods for additional analyses (e.g., subgroup analyses) {20b}

In an effort to conduct a robust, comprehensive evaluation of the PATH intervention, moderator and mediation analyses will be conducted following primary outcome analysis. Specifically, exploratory analyses will investigate baseline moderators of the direct effects of the PATH intervention on sustained viral suppression. Specifically, models will include the main effects of the study arm, moderator, and the moderator-by-study arm product term as predictors of the primary outcome in separate models for each moderator. The following moderators (subgroups) will be explored: (race ethnicity) Hispanic vs. Black participants, (substance use) reported using non-prescription stimulants/opioids at baseline vs. not, (sex as biological variable) males vs. females, (gender) men (cis and trans) vs. women (cis and trans) vs. nonbinary, (mode of transmission/risk group) men who have sex with men (or trans women who have sex with men) vs. heterosexual vaginal or anal sex vs. injection drug use, and the other plausible moderators. Causal mediation analysis using the potential outcomes (counterfactual) framework [68, 69] will be used to examine hypothesized mediators of intervention efficacy. It is hypothesized that even if there is an absence of a total effect of the intervention on the primary or secondary outcomes, there will be indirect effects of the PATH intervention (compared to control) through the mediators listed in Fig. 1.

To explore substance use-related outcomes, which is one of the study objectives, the same intention-to-treat approach will be applied to the primary outcome analyses. We will explore whether the PATH intervention (compared to control) significantly reduced the frequency of substance use (e.g., from using almost every day to once a week), drug cravings (urge for continued use), number of sex acts in the context of substance use in the past 3 months, and/or engagement in substance use treatment.

Other analyses will explore a dose-response effect of peer navigator factors and PATH webapp engagement. For peer navigator factors, we measure number of encounters between the participant and a peer navigator (e.g., via the PATH webapp, Zoom, phone), participant satisfaction with peer navigator and perceived rapport with peer navigator (measured in follow-up surveys); for PATH webapp engagement, we measure number of log-ins, proportion of days completing the ART adherence self-monitoring logs, number of views of HIV, ART and drug use “tips,” and number of posts in the social networking feed. Peer navigator factors and web app engagement will be treated as predictors of the outcomes in regression models. The analytic approach will follow those for our primary analysis, with regression models adjusting for any necessary baseline factors, and building multivariable models to examine independent associations.

### Methods in analysis to handle protocol non-adherence and any statistical methods to handle missing data {20c}

Outcome analysis on the primary and secondary outcomes will follow the intention-to-treat principle; thus, participants will be analyzed as they were randomized. Secondary per-protocol analyses on both the primary and secondary outcomes will also be conducted to examine potential outcome differences between the control group and subgroups of participants in the intervention arm who were more or less engaged in the intervention (e.g., high vs. low webapp engagement, high vs. low peer navigator engagement). Prior to this, the groups will be examined to assess for a potential imbalance between the groups, any characteristics for which there is an imbalance will be adjusted for during the secondary per protocol analyses. With regard to missing data, maximum likelihood will be used to address incomplete data because this makes the relatively mild assumption that missing data are conditionally missing at random. However, the missingness pattern will be diagnosed and an appropriate method to adjust for the missingness will be applied (e.g., inverse probability weighting, multiple imputation).

### Plans to give access to the full protocol, participant-level data, and statistical code {31c}

De-identified participant-level raw data and case report forms will be available upon reasonable request from the principal investigator once all outcome and secondary analyses have been completed and published. If cleaned and prepared datasets are not available, then statistical code to generate cleaned and prepared datasets will be available upon reasonable request from the principal investigator.

## Oversight and monitoring

### Composition of the coordinating center and trial steering committee {5d}

The trial is being implemented with the support of trained research staff, including a project manager who is responsible for monitoring study activities for the purpose of creating weekly recruitment and enrolment reports and a study coordinator who is based on-site at the community-based FQHC and is responsible for overseeing recruitment, enrolment, and follow-up survey collection. The study coordinator receives supervision and guidance from the Principal Investigators (including the Site Principal Investigator), a research supervisor at the community-based FQHC, and the HIV Clinical Services Manager at the community-based FQHC. Other staff include trained individuals who are bilingual and receive training in recruitment and/or enrolment. Peer navigators are not considered research staff and are not involved in recruitment, enrollment, and follow-up procedures with participants. Altogether, the investigative team is responsible for providing scientific guidance and oversight around the design and implementation of the trial. Data are being managed collectively by the project manager, study coordinator, Principal Investigator, and Site Principal Investigator.

The development and implementation of the study also received advisement from two pre-existing community advisory boards, one at the community-based FQHC (comprised mainly of patients living with HIV who have stayed retained in care and have maintained viral suppression) and the other is a board that provides advisement to HIV-related research studies focused on addressing health equity. The two boards provided input on the design of the trial and the PATH intervention, are given updates about the trial, and provide ongoing input on an annual basis.

### Composition of the data monitoring committee, its role, and reporting structure {21a}

A Data and Safety Monitoring Plan (DSMP) was developed in accordance with NIH policies, including the Policy for Data and Safety Monitoring dated June 10, 1998, and updated policy dated June 5, 2000. The monitoring of the DSMP is led by the principal investigator, site principal investigator, and the investigative team in collaboration with a Data Safety and Monitoring Board (DSMB) convened for the study. As described in Sect. 22 below, the principal investigator and site principal investigator are involved in responding to adverse events onsite at the community-based FQHC or in the office of the principal investigator. DSMB members are interdisciplinary researchers experienced with biomedical and behavioral interventions with marginalized populations including PLWH and people who use drugs. All DSMB members are external to the proposed project. The DSMB meets on an annual basis.

Any potentially adverse events are managed by the principal investigator and site principal investigator, with the support of the research team, and in consultation with the DSMB. Monitoring of potentially adverse events is a standing item on the research team’s weekly call. All potentially adverse events are documented, as are any resulting responses implemented by the study team. This information is compiled every 6 months by the research team into a DSMP report that is presented at the DSMB meetings and submitted alongside the annual IRB renewals and NIH progress reports.

As a clinical trial of a behavioral intervention, the PATH trial is registered in accordance with guidelines at clinicaltrials.gov. Final study data will be reported to this governing agency; the trial number is included in all progress reports, presentations, and publications. A summary report of findings, including adverse events, will be provided no later than 2 years after the completion date for the registered trial.

### Adverse event reporting and harms {22}

Participants are informed during the consent procedure that confidentiality may be breached under certain circumstances such as child abuse or threats of violence to the self or others. In cases where a breach of confidentiality is necessary (for example, due to mandated reporting of violence against a minor), project staff are instructed to report to the PI and Site PI, and the PI files an incident report with the study IRB within 24 h. All other potentially adverse events or serious problems are reported within 48 h on a standard form to the study IRB. Monitoring of potential adverse events or serious problems is given high priority by project staff and researchers. The incident report requires a detailed account of the problem, date of occurrence, date it came to the PI’s attention, impact on the participant, and corrective action taken. Serious adverse events (within 48 h) and unanticipated (non-serious) adverse events or problems (within 30 days) are reported by the PI and Site PI to the IRB. The IRB determines if the event was related to research, directly or indirectly, and, if so, requires revisions of protocol or consent, as applicable, and re-review of these procedures by the IRB. All project staff maintain their respective institution’s Human Subjects certification and certification in the Responsible Conduct of Research (RCR) to ensure that all staff are aware of the type of adverse events that may warrant a breach of confidentiality.

Both the PI and Site PI, and the IRB, are directly responsible for monitoring the security of the data and the safety of participants. All project staff report all potentially distressing adverse events (severity 2–3) to the Site PI, who is responsible for evaluating, internal reporting, and referring the participant to an appropriate professional. The PI and Site PI and the IRB are responsible for distinguishing a serious adverse event from a non-serious adverse event and an unanticipated problem. The PI and Site PI are responsible for immediately reporting any breaches of protocol, breakdowns in the consent process, violations of confidentiality of the data, complaints by participants, or any other serious problems or adverse events. Serious adverse events are reported to the PI and Site PI, the IRB, and the NIH program officer within 48 h. Following any serious adverse events or unanticipated problems, the PI and Site PI remain in contact with research staff in the 72-h period following the event to receive updates on the issue. If the issue is not resolved in that time frame, a follow-up report of the adverse event or unanticipated problem is given within 48 h to the IRB and NIH.

### Frequency and plans for auditing trial conduct {23}

Trial conduct is reviewed on a regular basis during the team’s weekly meetings, and as such are not independent from the investigators.

### Plans for communicating important protocol amendments to relevant parties (e.g., trial participants, ethical committees) {25}

Any study amendments are reviewed and approved by the Institutional Review Board (IRB) at San Diego State University prior to their implementation. Any important protocol modifications, such as changes to eligibility criteria, would be discussed and approved by all trial investigators and reported on the trial registry (clinicaltrials.gov).

## Dissemination plans {31a}

Results from the trial will be shared on the trial registry (clinicaltrials.gov) and will be disseminated via publications, abstracts submitted to scientific conferences, through oral presentations given to the community advisory boards providing consultation on the study, and through opportunities at other community forums (such as the city-wide weekly global health rounds). Study results will also be disseminated internally to staff at the community health center via staff meeting presentations. Finally, results will be shared with participants through mediums (e.g., infographic shared on email) identified as appropriate by the study community advisory boards.

## Discussion

The PATH intervention integrates peer navigation and mHealth technology into a unified, scalable intervention that could both strengthen the impact of peer navigation on HIV care outcomes among PLWH, and simultaneously reduce the amount of implementation resources needed to support peer navigation in clinical settings. Not unlike other studies focused on marginalized populations, the challenge that we anticipated and have encountered thus far is with recruitment [70]. We originally aimed to recruit and enroll at least 15–16 new participants each month across 24 months. Instead, the enrollment rate on average has remained at a steady 11–12 new participants per month. By definition, the population from which we are trying to recruit are individuals who are poorly engaged in HIV care, or who are at risk of falling out of care. Additionally, the trial faced the unique factors associated with a trial launch during a global pandemic. Recruitment began in November 2021, during historically high rates of COVID-19 due to the Omicron variant [71]. Another historical factor affecting recruitment was the outbreak of MPOX in the summer of 2022 [72]. However, we believe that recruitment has been successful overall given the fact that it has continued at a consistent rate in the context of these unprecedented environmental challenges. Thus, in August 2023, the decision was made to extend the recruitment period to 30 months (+ 6 months).

Study retention and loss to follow-up are other factors of concern, since this is known to potentially lead to biased results in RCTs [73, 74]. As described in our analytic approach, we will account for any characteristics associated with loss to follow-up during analysis. Prior to that, we are making every effort to promote retention and minimize loss to follow-up, with the goal of maintaining at least 80% retention at each study visit. We expect to meet this threshold, owing in large part to the dedicated staff overseeing study retention. These staff are not only highly trained but are also employed by the community-based FQHC where the trial is being conducted, potentially minimizing any distrust of researchers that is often found especially in marginalized and racial/ethnic minority communities [75, 76].

Community-based participatory or community-engaged research is held up as a best practice for research with marginalized or stigmatized communities who often face inequities in access to and retention in HIV prevention and care [77–83]. This study is a community-based RCT in a number of ways, including by receiving regular consultation from community advisory boards, and by being implemented in partnership with a community-based FQHC. The health center was founded in 1969 by a group of mothers with limited formal education who became interested in organizing their efforts with the vision of having the best care available for the most vulnerable in their community. The health center has grown significantly since then, but continues its focus on providing services to racially and ethnically diverse populations and has built trust in those communities. The fact that the PATH intervention is being evaluated in this setting should help to facilitate both efficacy and implementation outcomes, promoting the potential for longer-term sustainability if the intervention is found to be effective at improving the desired outcomes.

Finally, while the RCT described in this paper focuses on examining the efficacy of the PATH intervention, it is important to note that by design, the intervention is being evaluated in a real-world clinical setting. In order to take advantage of this opportunity to examine efficacy in a community-based FQHC, an ancillary goal is to also examine implementation factors and outcomes. Thus, although not technically registered as such, the activities in this RCT mirror what would be seen in a hybrid type I effectiveness-implementation trial, which has the primary aim to examine the effectiveness of the intervention, and a secondary aim to better understand the context for implementation (e.g., potential barriers and facilitating factors) [84]. Specifically, while adhering to the RCT protocol, we are exploring implementation barriers and facilitators of the PATH intervention being implemented in a community-based FQHC. Informed by established implementation science frameworks [85, 86], which for example outline the roles of organizational (e.g., facilitative administration, decision support data system) and competency (e.g., staff selection, training) drivers in affecting implementation [85], and the idea that movement across the implementation process is affected by such factors [86], we are conducting in-depth qualitative interviews with PATH peer navigators, supervisors, the research study staff, and staff at the community-based FQHC (e.g., case managers). Though exploratory and secondary to the main goals of the study, these data will provide value-added to the existing study by shedding light on the implementation context of the PATH intervention. The implementation-related data may inform a future trial focusing on implementation outcomes (e.g., feasibility, acceptability, sustainability, cost-effectiveness) [87] of PATH in other health clinics offering the Ryan White Program model of care for PLWH.

## Trial status

This is protocol version 1.0. Recruitment for the trial began on November 4, 2021. It is estimated that recruitment will be completed in June 2024.

## Data Availability

The final trial dataset will be available upon reasonable request from the principal investigator.

## Abbreviations

HIV: Human immunodeficiency virus
PATH: Peers plus mobile app for treatment in HIV
ART: Antiretroviral therapy
FQHC: Federally Qualified Health Center
UC: Usual care
IRB: Institutional Review Board
